# Cross-cultural adaptation of the Richmond Agitation-Sedation Scale to Brazilian Portuguese for the evaluation of sedation in pediatric intensive care

**DOI:** 10.5935/0103-507X.20210011

**Published:** 2021

**Authors:** Letícia Massaud-Ribeiro, Maria Clara de Magalhães Barbosa, Anderson Gonçalves Panisset, Jaqueline Rodrigues Robaina, Fernanda Lima-Setta, Arnaldo Prata-Barbosa, Antonio José Ledo Alves da Cunha

**Affiliations:** 1 Instituto de Puericultura e Pediatria Martagão Gesteira, Universidade Federal do Rio de Janeiro - Rio de Janeiro (RJ), Brazil.; 2 Instituto D’Or de Pesquisa e Ensino - Rio de Janeiro (RJ), Brazil.

**Keywords:** Translating, Cross-cultural comparison, Conscious sedation, Deep sedation, Intensive care units, pediatric, Tradução, Comparação transcultural, Sedação consciente, Sedação profunda, Unidade de terapia intensiva pediátrica

## Abstract

**Objective:**

To perform a cross-cultural adaptation of the Richmond Agitation-Sedation Scale (RASS) to Brazilian Portuguese for the evaluation of sedation in pediatric intensive care.

**Methods:**

Cross-cultural adaptation process including the conceptual, item, semantic and operational equivalence stages according to current recommendations.

**Results:**

Pretests, divided into two stages, included 30 professionals from the pediatric intensive care unit of a university hospital, who administered the translated RASS to patients aged 29 days to 18 years. The pretests showed a content validity index above 0.90 for all items: 0.97 in the first stage of pretests and 0.99 in the second.

**Conclusion:**

The cross-cultural adaptation of RASS to Brazilian Portuguese resulted in a version with excellent comprehensibility and acceptability in a pediatric intensive care setting. Reliability and validity studies should be performed to evaluate the psychometric properties of the Brazilian Portuguese version of the RASS.

## Abstract (pt)

**Objetivo:**

Realizar adaptação transcultural para o Brasil da *Richmond Agitation-Sedation Scale* (RASS) para avaliação da sedação em terapia intensiva pediátrica

**Métodos:**

Processo de adaptação transcultural incluindo as etapas de equivalência conceitual, de itens, semântica e operacional, de acordo com recomendações atuais.

**Resultados:**

Pré-testes, divididos em duas etapas, incluíram 30 profissionais da unidade de terapia intensiva pediátrica de um hospital universitário, que aplicaram a RASS traduzida em pacientes de 29 dias a 18 anos. Os pré-testes mostraram Índice de Validade de Conteúdo acima de 0,90 para todos os itens: 0,97 na primeira etapa de pré-testes e 0,99 na segunda.

**Conclusão:**

A adaptação transcultural da RASS para o português do Brasil resultou em versão com excelente compreensão e aceitabilidade em cenário de terapia intensiva pediátrica. Estudos de confiabilidade e de validade devem ser realizados para avaliar as propriedades psicométricas da versão adaptada para o português do Brasil da RASS.

## INTRODUCTION

Sedation, like analgesia, covers a spectrum of states from minimal sedation to general anesthesia.^(1)^ In severely ill children, the administration of medications that induce sedation and analgesia is often necessary to promote comfort and pain relief, establish patient-ventilator synchrony during mechanical ventilation (MV) and prevent the accidental removal of devices necessary to maintain life, in addition to reducing the anxiety and discomfort associated with treatment in the intensive care setting.^(2)^

Similarly, inadequate or insufficient sedation can cause asynchrony during MV, inadvertent removal of catheters and drains, falls from bed and aggressive behavior of the patient towards the healthcare team.^(3)^ Patient stress can also result from insufficient sedation or analgesia and appears to be associated with increased myocardial oxygen consumption, hypercoagulability and immunosuppression.^(4-6)^ On the other hand, the use of sedatives or analgesics in high doses and for long periods is associated with several adverse events and unfavorable outcomes, such as increases in the duration of MV,^(7,8)^ incidence of pneumonia,^(9)^ occurrence of delirium and withdrawal syndrome,^(10)^ length of hospital stay and mortality^(11)^ and hospitalization costs.^(12,13)^ It also leads to reduced bed mobility and increased thromboembolic phenomena, muscle weakness and skin lesions.^(4)^

Several recommendations for good clinical practice emphasize the need for goal-directed therapy in the administration of sedatives and advocate the inclusion of this approach as standard care in intensive care units (ICUs) due to the impact of the use of sedatives on the discontinuation of ventilatory support and the length of stay in the ICU.^(3,4)^ In pediatric patients, the association between the use of sedation protocols and decreased duration of MV, length of ICU stay, frequency of unplanned extubation and prevalence of withdrawal syndrome has been demonstrated.^(14-16)^ Additionally, in children with acute respiratory distress syndrome, there is a strong recommendation to offer minimal but effective goal-directed sedation through the use of protocols to monitor, titrate and guide sedation.^(17)^ To follow the recommendations of goal-directed therapy for both adults and children, it is essential to use an instrument to measure the level of sedation.

There are several subjective scales for assessing sedation and agitation in adults admitted to the ICU. Of 10 scales reviewed for their psychometric properties, the Richmond Agitation-Sedation Scale (RASS) and the Sedation-Agitation Scale (SAS) were considered to have the best validity and reliability for measuring the quality and depth of sedation in adults; they are useful even for evaluating patient response, even in those who are not receiving a continuous infusion of sedation/analgesia drugs. Additionally, the RASS consistently provides a consensual target for goal-directed sedative therapy and is the only instrument to demonstrate viability of use and clinical relevance.^(18)^ Recently, the RASS underwent a validation study in children with and without the use of sedatives and analgesics and showed good performance.^(19)^ In Brazil, it has been translated into Brazilian Portuguese,^(20)^ but a complete cross-cultural adaptation was not performed.

The objective of this study was to perform a cross-cultural adaptation of the RASS to Brazilian Portuguese according to the current recommendations.^(21,22)^

## METHODS

First, authorization was obtained from the creators of the original RASS, which is copyrighted by Virginia Commonwealth University in Richmond, Virginia, in the United States.^(23)^ The cross-cultural adaptation followed the universalist approach of Herdman et al.^(21)^ and Reichenheim and Moraes,^(24)^ which evaluates six equivalences: conceptual, item, semantic, operational, measurement and functional. These steps are similar to those recommended by the Translation and Cultural Adaptation Process for Patient-Reported Outcomes-Principles of Good Practice of the International Society for Pharmacoeconomics and Outcomes Research (ISPOR),^(22)^ although the terms used to designate each step differ. The present study focused only on conceptual, item, semantic and operational equivalences and did not evaluate the measurement and functional equivalencies.

A literature review and meetings with six specialists in pediatrics or pediatric intensive care took place during the evaluation of conceptual and item equivalences. For the evaluation of semantic equivalence, three independent translations of the RASS from English into Brazilian Portuguese were performed by three Brazilian physicians specializing in pediatric intensive care who were fluent in English. These translations were reconciled into a preliminary version through a consensus meeting with the three translators and other specialists from the research team. The reconciled translation underwent backtranslation into English by a North American translator fluent in Brazilian Portuguese who did not receive prior information about the instrument. This translator provided two backtranslations: a literal one and a more conceptual version. The equivalence between these versions and the original instrument was reexamined in a new round of discussions among the specialists to reconcile them into a single backtranslation, which was sent to the author of the original instrument for approval. The entire translation, reconciliation and backtranslation process included the RASS itself and the instructions for its application, which are part of the instrument. To complement the evaluation of semantic equivalence and to evaluate operational equivalence, pretests were performed in two stages, in which the reconciled version of the instrument was administered by 30 health professionals (physicians, nurses and physical therapists) to patients admitted to the pediatric ICU of the *Instituto de Puericultura e Pediatria Martagão Gesteira* of the *Universidade Federal do Rio de Janeiro* (UFRJ). The inclusion criteria were age 29 days to 18 years old, and the exclusion criteria were neuromuscular blockade use, quadriplegia, previous diagnosis of reduced auditory or visual acuity or suspected or confirmed brain death. Each professional participating in the pretest received a form showing RASS version, formatted as in the original studies, plus a space next to each item showing five Likert-type response options (1 - “I did not understand anything”; 2 - “I understood a little”; 3 - “I understood part of it”; 4 - “I understood almost everything, but I had some doubts”; 5 - “I understood it perfectly, and I had no doubts”), and the professional chose the answer that best characterized their understanding of the respective item. There were also open fields where the professionals could add questions and suggestions. The responses and suggested modifications were discussed and reconciled at two consensus meetings, one after the first pretest stage (first 16 pretests) and the other after the second pretest stage (14 more pretests). These meetings results in the final version.

The pretest results were evaluated by calculating the median Likert-type responses regarding the comprehensibility of each item and the content validity index (CVI) for the adaptation of the measurement instrument. The CVI measures the percentage of evaluators who agree with a particular item and allows the assessment of both each item alone and the instrument as a whole. The CVI of each item was calculated by dividing the sum of the number of 4 or 5 responses (“I understood almost everything, but I had some doubts” and “I understood perfectly and I had no doubts”, respectively) for a given item by the total number of responses for that item. The total CVI was calculated as the mean of the CVIs of each item.^(25)^ The minimum agreement suggested when the evaluation is performed by six or more subjects is 0.78.^(26)^ Excel 2010 software (Microsoft Corporation) was used for data entry and analysis. Informed consent forms were signed by the professionals and the guardians of the patients who participated in the pretest. The study was approved by the Research Ethics Committee of the *Instituto de Puericultura e Pediatria Martagão Gesteira* of UFRJ under opinion 2.553.042.

### Description of the Richmond Agitation-Sedation Scale

The RASS comprises ten items that describe gradual levels of agitation/sedation, their respective scores, and instructions for administering the instrument and is into three steps. The first step is to observe the patient. If the patient is alert and calm, a score of zero should be assigned. If the patient presents behavior compatible with restlessness or agitation, a score from +1 to +4 should be assigned according to the descriptions of different levels of agitation in the “Description” column of the scale. If the patient is not alert, the professional should proceed to the second step and call the patient by name. If the patient opens his/her eyes and maintains eye contact for more than 10 seconds, a score of -1 should be assigned. If eye contact is not maintained for more than 10 seconds, a score of -2 should be assigned. If the patient shows any movement in response to verbal command but does not make eye contact, a score of -3 should be assigned. If the patient does not respond to the verbal command, the third step is taken, which consists of physically stimulating the patient. If the patient shows any movement in response to the physical stimulus, a score of -4 should be assigned. Finally, if the patient does not show any response to verbal commands or physical stimuli, a score of -5 should be assigned. If the patient is not alert before the verbal command or the physical stimulus is given, a score of -1 to -5 (according to the “Description” column of the scale) should be assigned, even if the patient becomes agitated after the stimulus. The author of the original scale reported an application time of less than 20 seconds.^(27)^

## RESULTS

The reconciled backtranslation into English was approved by the author of the original RASS, Curtis N. Sessler, on May 25, 2017. The professionals participating in the pretest had the following distribution according to their professional training: 15 (50%) were physicians, 12 (40%) were nurses, and three (10%) were physical therapists. Regarding the patients who participated in the pretest (n = 8), the median age was 6 months (interquartile range: 1 - 59), and the most frequent diagnosis was respiratory diseases (Table 1). In seven of the 30 pretests, the patients were receiving continuous infusion of at least one of the following sedatives or analgesics: midazolam, fentanyl or dexmedetomidine.

**Table 1:** Demographic and clinical characteristics of patients in the pretest of the Richmond Agitation-Sedation Scale translated into Brazilian Portuguese (n = 8)

Characteristics	
Age (months)	6 (1 - 59)
Sex	
Female	3 (37.5)
Male	5 (62.5)
Diagnosis	
Neurological	1 (12.5)
Oncohematological	2 (25.0)
Respiratory	5 (62.5)
RASS	-2.5 (-4 - 0)

RASS - Richmond Agitation-Sedation Scale. The results are expressed as n (%) or median (interquartile range).

In 27 of the 30 pretests, all items received responses denoting a good degree of understanding, corresponding to scores of 4 and 5 on the Likert scale (“I understood almost everything, but I had some doubts” and “I understood perfectly, and I had no doubts”, respectively). The CVI of all items was above 0.90, as was the total CVI for the two pretest stages (Table 2). After the first stage, which included the first 16 of the 30 pretests, three changes were made to the text of the instrument: after the term “*Procedimento*”, the phrase “*para aplicação da escala de agitação e sedação de Richmond*” was added; in step 1 of the Procedure, the term “*consistente*” was replaced with “*compatível*”; and in step 2 of the Procedure, the term “*exceto*” was replaced by “*mas sem*”. In addition to these changes, a change was made to the table format; a model was adopted that was similar to that used in the reliability and validity studies of the original instrument,^(27)^ which specified, in a column to the right of the items referring to the different levels of sedation, the verbal command or physical stimulus that should be performed and indicated in the “*Procedimento*” section the correspondence between the observed responses and the score to be assigned.

**Table 2:** Results of the two pretest stages, in which professionals from the pediatric intensive care unit team assessed the comprehensibility of the Richmond Agitation-Sedation Scale translated into Brazilian Portuguese

Item	First stage (n = 16)		Second stage (n = 14)	
	Median (IQ 25 - 75)	CVI	Median (IQ 25 - 75)	CVI
Scale				
Score +4	5 (4.3 - 5)	0.9375	5 (5 - 5)	1
Score +3	5 (5 - 5)	0.9375	5 (4.8 - 5)	1
Score +2	5 (5 - 5)	0.9375	5 (5 - 5)	1
Score +1	5 (5 - 5)	1	5 (5 - 5)	1
Score 0	5 (5 - 5)	1	5 (5 - 5)	1
Score -1	5 (5 - 5)	1	5 (5 - 5)	0.9285
Score -2	5 (5 - 5)	1	5 (5 - 5)	1
Score -3	5 (5 - 5)	0.9375	5 (5 - 5)	1
Score -4	5 (5 - 5)	1	5 (5 - 5)	1
Score -5	5 (5 - 5)	1	5 (5 - 5)	1
Instructions				
First step	5 (5 - 5)	0.9375	5 (5 - 5)	1
Second step	5 (5 - 5)	0.9375	5 (5 - 5)	1
Third step	5 (5 - 5)	0.9375	5 (5 - 5)	1
Total		0.9708		0.9952

IQ - interquartile range; CVI - content validity index. Note: Of the 30 evaluations, seven were performed with the patient receiving a continuous infusion of midazolam, fentanyl and/or dexmedetomidine.

At the end of the second stage, which included 14 additional pretests, another format change was made: in the column to the right of the items referring to the different levels of agitation, it was indicated that observation of the patient alone was sufficient for classification. The word “*Procedimento*” was changed to “*Instruções*”, and the formatting of this section was changed again to incorporate the scores to be assigned for each level of agitation/sedation into the text instead of presenting them in a separate column (Table 3 and Figure 1). In the comments field of the form used for the pretest, the following terms were questioned by at least one of the participants: “*combativo*”, “*agressivo*”, “*sem propósito*”, “*não totalmente alerta*”, “*qualquer movimento*” and “*consistente*”. In addition, two participants questioned the equivalence of the item with a score of +4 (“*Claramente combativo ou violento; perigo imediato para a equipe”)* because it seemed inappropriate for use with infants, who would hardly reach a score of +4. In this same context, a participant also questioned the feasibility of applying Step 2 in very young infants because it required the patient to make eye contact upon being called by his/her name by the examiner, which is not compatible with normal development in that age group.

**Table 3:** Comparison of changed items in the Brazilian Portuguese versions of the Richmond Agitation-Sedation Scale

Item	Primeira versão	Segunda versão	Versão final
Escore +2	Agitado - movimentos sem propósito frequentes ou assincronia paciente-ventilador	Agitado - movimentos frequentes sem objetivo ou assincronia paciente-ventilador	Agitado - movimentos frequentes sem objetivo ou assincronia paciente-ventilador
Escore -1	Sonolento - não totalmente alerta, mas mantém despertar sustentado (mais de 10 segundos), com contato visual, ao comando verbal	Sonolento - não totalmente alerta ao comando verbal, mas mantém despertar sustentado (mais de 10 segundos), com contato visual	Sonolento - não totalmente alerta ao comando verbal, mas mantém despertar sustentado (mais de 10 segundos), com contato visual
Escore -2	Sedação leve - desperta brevemente (menos de 10 segundos), com contato visual, ao comando verbal	Sedação leve - ao comando verbal, desperta brevemente (menos de 10 segundos), com contato visual	Sedação leve - ao comando verbal, desperta brevemente (menos de 10 segundos), com contato visual
Escore -3	Sedação moderada - qualquer movimento (mas sem contato visual) ao comando verbal	Sedação moderada - qualquer movimento ao comando verbal (mas sem contato visual)	Sedação moderada - qualquer movimento ao comando verbal (mas sem contato visual)
Primeiro passo	1. Observe o paciente. O paciente está alerta e calmo (escore 0)? O paciente tem um comportamento consistente com inquietação ou agitação (escore +1 a +4, usando os critérios listados na coluna "Descrição" da escala (Figura 1)?	1. Observe o paciente O paciente está alerta e calmo O paciente tem um comportamento compatível com inquietação ou agitação	1. Observe o paciente. Se o paciente estiver alerta e calmo, atribua escore 0 Se o paciente estiver com um comportamento compatível com inquietação ou agitação, atribua escore de +1 a +4, de acordo com a coluna "Descrição" da escala (Figura 1)
Segundo passo	2. Se o paciente não estiver alerta, chame-o pelo nome, em voz alta, mande-o abrir os olhos e olhar para você. Repita uma vez, se necessário. O paciente pode ser verbalmente estimulado a continuar olhando para você, mas você não deve tocá-lo ou estimulá-lo fisicamente. O paciente apresenta abertura ocular e contato visual, mantido por mais de 10 segundos (escore -1). O paciente apresenta abertura ocular e contato visual mantido por não mais do que 10 segundos (escore -2). O paciente apresenta qualquer movimento em resposta ao comando verbal, exceto contato visual (escore -3)	2. Se o paciente não estiver alerta, chame-o pelo nome, em voz alta, mande-o abrir os olhos e olhar para você. Repita uma vez, se necessário. O paciente pode ser verbalmente estimulado a continuar olhando para você, mas você não deve tocá-lo ou estimulá-lo fisicamente O paciente apresenta abertura ocular e contato visual, mantido por mais de 10 segundos O paciente apresenta abertura ocular e contato visual mantido por não mais do que 10 segundos O paciente apresenta qualquer movimento em resposta ao comando verbal, mas sem contato visual	2. Se o paciente não estiver alerta, chame-o pelo nome, em voz alta, mande-o abrir os olhos e olhar para você. Repita uma vez, se necessário. O paciente pode ser verbalmente estimulado a continuar olhando para você, mas você não deve tocá-lo ou estimulá-lo fisicamente Se o paciente apresentar abertura ocular e contato visual, mantido por mais de 10 segundos, atribua escore -1 Se o paciente apresentar abertura ocular e contato visual mantido por não mais do que 10 segundos, atribua escore -2 Se o paciente apresentar qualquer movimento em resposta ao comando verbal, mas sem contato visual, atribua escore -3
Terceiro passo	3. Se o paciente não responder ao comando verbal, estimule-o fisicamente sacudindo seu ombro e, em seguida, friccionando seu esterno, caso não haja resposta ao estímulo no ombro. O paciente apresenta qualquer movimento ao estímulo físico (escore -4). O paciente não apresenta qualquer resposta ao comando verbal ou ao estímulo físico (escore -5)	3. Se o paciente não responder ao comando verbal, estimule-o fisicamente sacudindo seu ombro e, em seguida, friccionando seu esterno, caso não haja resposta ao estímulo no ombro O paciente apresenta qualquer movimento ao estímulo físico O paciente não apresenta qualquer resposta ao comando verbal ou ao estímulo físico	3. Se o paciente não responder ao comando verbal, estimule-o fisicamente sacudindo seu ombro e, em seguida, friccionando seu esterno, caso não haja resposta ao estímulo no ombro Se o paciente apresentar qualquer movimento ao estímulo físico, atribua escore -4 Se o paciente não apresentar qualquer resposta ao comando verbal ou ao estímulo físico, atribua escore -5

**Figure 1:** Final Brazilian Portuguese version of the Richmond Agitation-Sedation Scale.

**Escala de agitação e sedação de Richmond** Antes de aplicar a RASS, leia as instruções da escala			
**Escore**	**Termo**	**Descrição**	
+4	Combativo	Claramente combativo ou violento: perigo imediato para a equipe	Observação do paciente
+ 3	Muito agitado	Puxa ou remove tubo(s) ou cateter(es) ou apresenta comportamento agressivo com a equipe	
+2	Agitado	Movimentos frequentes sem objetivo ou assincronia paciente-ventilador	
+1	Inquieto	Ansioso ou apreensivo, porém sem movimentos agressivos ou vigorosos	
0	Alerta e calmo		
-1	Sonolento	Não totalmente alerta ao comando verbal, mas mantém despertar sustentado (mais de 10 segundos), com contato visual	Comando verbal
-2	Sedação leve	Ao comando verbal, desperta brevemente (menos de 10 segundos), com contato visual	
-3	Sedação moderada	Qualquer movimento ao comando verbal (mas sem contato visual)	
-4	Sedação profunda	Sem resposta ao comando verbal, mas com qualquer movimento ao estímulo físico	Estímulo físico
-5	Não reponde a estímulos	Sem resposta ao comando verbal ou ao estímulo físico	
**Instruções para aplicação da escala de agitação e sedação de Richmond**			
1.	Observe o paciente		
	Se o paciente estiver alerta e calmo, atribua **escore 0**		
	Se o paciente estiver com um comportamento compatível com inquietação ou agitação, atribua **escore de +1 a + 4**, de acordo com a coluna "Descrição" da escala		
2.	Se o paciente não estiver alerta, chame-o pelo nome, em voz alta, mande-o abrir os olhos e olhar para você. Repita uma vez, se necessário. O paciente pode ser verbalmente estimulado a continuar olhando para você, mas **você não deve tocá-lo ou estimulá-lo fisicamente**		
	Se o paciente apresentar abertura ocular e contato visual, mantido por mais de 10 segundos, atribua **escore -1**		
	Se o paciente apresentar abertura ocular e contato visual, mantido por não mais do que 10 segundos, atribua **escore -2**		
	Se o paciente apresentar qualquer movimento em resposta ao comando verbal, mas sem contato visual, atribua **escore -3**		
3.	Se o paciente não responder ao comando verbal, estimule-o fisicamente sacudindo seu ombro e, em seguida, friccionando seu esterno, caso não haja resposta ao estímulo no ombro		
	Se o paciente apresentar qualquer movimento ao estímulo físico, atribua **escore -4**		
	Se o paciente não apresentar qualquer resposta ao comando verbal ou ao estímulo físico, atribua **escore -5**		

The mean (standard deviation) and median (interquartile range) RASS scores assigned by the professionals were -2.1 (2) and -2.5 (-4 - 0), respectively. The final version of the scale in Brazilian Portuguese, including the results of the evaluation of conceptual, item, semantic and operational equivalences, is shown in figure 1.

## DISCUSSION

This is the first cross-cultural adaptation study of the RASS for Brazilian Portuguese to be conducted according to international recommendations to ensure the quality of the results. The final version of the RASS adapted for Brazilian Portuguese showed evidence of good conceptual, item, semantic and operational equivalences. In 2008, Nassar Júnior et al.^(20)^ conducted a reliability and validity study of four sedation scales used in intensive care based on a Brazilian Portuguese version of the RASS, but they did not describe how and whether translation and cross-cultural adaptation processes were performed. Apparently, these authors main focus was reporting the reliability and validity of the scales. For this reason, we considered it pertinent to conduct this study, which describes, in detail, the entire process of translating and cross-culturally adapting the scale to Brazilian Portuguese.

The scarcity of instruments for assessing the level of sedation properly adapted for use in pediatric ICUs in Brazil may compromise the quality of care by facilitating the occurrence of adverse events and unfavorable outcomes associated with the use of nonoptimal levels of sedation. However, the use of a measurement instrument in a cultural context different from its original context requires a detailed process of cross-cultural equivalence to be considered safe. Often, the change in context presupposes not only translation into another language but also adaptations that may be necessary for use in another country with the same language or even in different regions of a country.^(28)^ Failures in this process can compromise the application of the instrument and hinder future comparisons. In this study, we sought to follow all the steps of a formal process of cross-cultural adaptation for measurement instruments.

In our study, the main changes made to the instrument after the two pretest stages were operational and related to the formatting of the instrument (with the insertion of a column relating the type of stimulus performed to the degree of agitation/sedation) and the reinforcement of the information related to the instructions for application of the instrument. These changes reflect some degree of difficulty, albeit small, in the application of the instrument by health professionals, which we aim to remedy with the use of more explicit instructions. Part of these formatting changes had already been performed in the validation of the instrument in its original language.^(27)^ There were few semantic changes to the items of the scale.

The observations made freely by the pretest participants contributed later in the consensus meetings to an understanding of the doubts reported in the pretest evaluations. All observations were discussed with the research team, and some modifications made by the participating professionals were incorporated into the final version of the instrument. The most relevant were those related to the application of the instrument in infants, which showed limitations for application in this age group. The authors of a recent study also identified this limitation and adapted the instrument in its original language for levels -2 and -3, adding “eye opening” in newborns as a response equivalent to “eye contact” in older children.^(29)^ Thus, a score of -2 was defined as “Briefly awakens with eye contact in response to voice (< 10 seconds)”, and a score of -3 was defined as “Movement or eye opening in response to voice (but no eye contact)”. The adaptation of the scale for infants has not yet been validated and, in our opinion, does not seem to offer good discrimination between these two levels.

There is great variability in therapeutic practices and sedation assessment for children admitted to the ICU,^(30)^ which can make it difficult to obtain an optimal level of sedation.^(31,32)^ Many ICUs do not use any instrument for such evaluations.^(33,34)^ In children, the most commonly used scales are the Comfort, Comfort-Behavior and State Behavior Scale.^(35-37)^ All were validated in children, but none was superior.^(2)^ In Brazil, however, a recent study among intensivist pediatricians revealed that the most commonly used scale is the Ramsay scale.^(38)^

The Comfort scale is the most widespread in pediatric ICUs in several countries.^(39,40)^ Although it was designed to assess discomfort in children, it was operationalized to include both the agitation and pain constructs. The use of a specific instrument for grading agitation/sedation would allow a more targeted therapeutic adjustment.^(41)^ In addition, the Comfort scale uses behavioral and physiological variables. Because the latter may be controlled in patients admitted to the ICU; their usefulness was questioned, resulting in the development of the Comfort-Behavior scale, which uses only behavioral variables, in addition to an item related to crying to evaluate children no longer on mechanical ventilation. Amoretti et al.^(42)^ performed the translation and backtranslation of the Comfort-Behavior scale into Brazilian Portuguese and conducted a validation study, but this scale is considered complex due to the number of variables at each level and is quite extensive.^(43)^ The Ramsay scale, in turn, is a simple scale that provides some discrimination among the levels of sedation but only describes one level of agitation. The RASS, in contrast, is a specific scale for evaluating both sedation and agitation and can be applied to patients both with and without the use of sedatives or analgesics. It describes 10 levels of consciousness ranging from extreme agitation to deep sedation, and it can be applied quickly according to three well-defined steps. In addition to being easy to use, it has been shown to have excellent interobserver reliability in adults admitted to clinical and surgical ICUs and excellent validity when compared to the VAS and other selected sedation scales (Glasgow Coma Scale, Ramsay and SAS).^(23,27)^ It has been widely used not only to grade the level of sedation or agitation for monitoring and therapeutic adjustment but also as a prerequisite for the application of other instruments, such as the Pediatric Confusion Assessment Method for the Intensive Care Unit (pCAM-ICU)^(44)^ and the Cornell Assessment of Pediatric Delirium,^(45)^ both of which have been adapted for Brazilian Portuguese,^(46,47)^ the Preschool Confusion Assessment Method for the ICU (psCAM-ICU)^(48)^ and the Sophia Observation Withdrawal Symptoms scale-Pediatric Delirium scale (SOS-PD).^(49)^ The RASS has undergone cross-cultural adaptation for other languages, such as Swedish, Spanish and Serbian.^(50-52)^ We chose to perform a cross-cultural adaptation of RASS because of its simplicity, objectivity and rapid application.

We can identify some limitations in this study. Only one translator was responsible for the backtranslation, although he provided two versions, one literal and one conceptual. However, several specialists were involved in the other stages of semantic equivalence, including the performance of three independent translations from English to Portuguese, which allowed differences in translation to be compared and the best terms to be chosen for the reconciled version. Another limitation was the small number of patients with positive scores (or agitation) on the scale during the pretest evaluations; furthermore, no patients with extreme agitation (scores +3 and +4) were included. This fact can also be observed in the validation study of the original instrument in children,^(19)^ which, although it included a larger number of patients, did not include any patients with a score of +4. Another more recent study with an even larger sample also obtained a similar distribution.^(29)^ It is possible that the scarcity of children with a score of +4 is due to an actual low frequency of children who present an extreme degree of agitation in the ICU. However, the inadequacy of this item for the infant age group, which represents a considerable portion of the pediatric population admitted to the ICU, may have contributed to this finding.

The available evidence for scales assessing the depth and quality of sedation in adults admitted to the ICU suggests that the RASS has a better psychometric performance than others. We believe that, with some adjustments for the infant age range, the instrument can maintain better psychometric performance than other scales for children of any age group. Adjustment of the Brazilian Portuguese version of the RASS for use with infants and the assessment of its validity and reliability in children admitted to the ICU are needed to corroborate the psychometric properties of the instrument for monitoring sedation in critically ill children of any age group.

## CONCLUSION

The cross-cultural adaptation of the RASS for Brazilian Portuguese was performed according to the current recommendations and resulted in a Brazilian version that should enable the performance of reliability and validity studies in children and adults in Brazil. However, its use in infants requires specific adaptations for that age group. Once the good psychometric performance of the adapted instrument has been demonstrated, the scale may meet the need for a simple and quickly applied scale to evaluate the depth and quality of sedation in any age group.
